# Variation in the prion protein gene (*PRNP*) open reading frame sequence in French cervids

**DOI:** 10.1186/s13567-024-01362-2

**Published:** 2024-09-03

**Authors:** Johann Laubier, Anne Van De Wiele, Aurélie Barboiron, Denis Laloë, Christine Saint-Andrieux, Johan Castille, Emma Meloni, Sonja Ernst, Maryline Pellerin, Sandrine Floriot, Nathalie Daniel-Carlier, Bruno Passet, Joël Merlet, Hélène Verheyden, Vincent Béringue, Olivier Andréoletti, Fiona Houston, Jean-Luc Vilotte, Vincent Bourret, Katayoun Moazami-Goudarzi

**Affiliations:** 1 INRAE, AgroParisTech, GABI, University Paris-Saclay, Jouy-en-Josas, France; 2 Research and Scientific Support Department, French Biodiversity Agency (OFB), Vincennes, France; 3 Friedrich-Loeffler-Institut, Isle of Riems, Germany; 4 INRAE, CEFS, Toulouse University, Castanet Tolosan, France; 5 INRAE, UVSQ, VIM, University Paris-Saclay, Jouy-en-Josas, France; 6 UMR INRAE ENVT 1225, IHAP, École Nationale Vétérinaire de Toulouse, Toulouse, France; 7 Division of Immunology, The Roslin Institute, Royal Dick School of Veterinary Studies, University of Edinburgh, Edinburgh, UK

**Keywords:** CWD, *PRNP*, polymorphism, roe deer, red deer, France

## Abstract

The recent emergence of chronic wasting disease (CWD) in Europe has become a new public health risk for monitoring of wild and farmed cervids. This disease, due to prions, has proliferated in North America in a contagious manner. In several mammalian species, polymorphisms in the prion protein gene (*PRNP*) play a crucial role in the susceptibility to prions and their spread. To obtain a reliable picture of the distribution of *PRNP* polymorphisms in the two most common cervid species in France, we sequenced the open reading frame (ORF) of this gene in 2114 animals, 1116 roe deer (*Capreolus capreolus*) and 998 red deer (*Cervus elaphus*). Selection criteria such as historical origin, spatial distribution and sex ratio have been integrated to establish this sample collection. Except for one heterozygous animal with a non-synonymous mutation at codon 37 (G37A), all the 1116 French roe deer were monomorphic. Red deer showed greater variation with two non-synonymous substitutions (T98A; Q226E), three synonymous substitutions (codons 21, 78 and 136) and a new 24pb deletion (Δ_69-77_). We found significant regional variations between French regions in the frequency of the identified substitutions. After cloning of the *PRNP* ORF from animals presenting multiple non-synonymous polymorphisms, we identified six haplotypes and obtained a total of twelve genotypes. As in other European countries, we highlighted the apparent homogeneity of *PRNP* in the French roe deer and the existence of a greater diversity in the red deer. These results were in line with European phylogeographic studies on these two species.

## Introduction

Chronic wasting disease (CWD) is a fatal transmissible spongiform encephalopathy (TSE) that affects captive and wild *Cervidae*. CWD has the widest potential species range among prion diseases and is the only one of greatest concern affecting wildlife populations. As such, it is recognised as a major emerging threat to wildlife. As for other TSE, CWD is caused by misfolding of the cellular prion protein (PrP^C^) to a pathogenic conformer (PrP^Sc^). CWD prions are often lymphotropic, at least in North American cervid species, and infected cervids likely shed prions replicated in lymphoid organs. Thus, CWD is mainly transmitted horizontally, through either direct contact or prion-contaminated environmental sources (e.g. remains of infectious carcasses, body fluids, feed, water, soil, fomites). Infection of naïve deer introduced to highly contaminated pens indicates that infectivity is retained for at least five years [1]. The species host range and thus zoonotic risks associated with CWD remains to be clearly established [2–7]. At the same time, it has been estimated that up to 15 000 CWD-infected cervids are consumed by people each year in the United States [2].

The disease’s range extends across North America and Northern Europe. It was first described in a mule deer (*Odocoileus hemionus*) at a research centre in Colorado in 1967 and in wild deer in 1981 but recent studies have suggested that CWD had already been present for ten to 20 years prior to its initial identification [8]. The disease is now geographically widespread in North America, being detected in 34 US states and five Canadian provinces in free-ranging cervids and/or commercial captive cervid facilities (United States Geological survey, National Wildlife Health Center, Updated June 2024). In heavily affected areas of Wyoming, Colorado and Wisconsin, more than 40% of free-ranging cervids are infected and population declines associated with CWD in white-tailed deer (*Odocoileus virginianus*), mule deer and elk (*Cervus elaphus nelsoni*) are documented [9–11].

In Europe, the first cases of infected wild reindeer (*Rangifer tarandus tarandus*) were detected in the mountainous region of Nordfjella in Norway in 2016 leading to the eradication of the entire population of around 2000 reindeer in the area [12]. Since then and up to December 2023, a total of 43 CWD cases were confirmed in Norway, Sweden and Finland, including moose (*Alces alces*), reindeer as well as red deer (*Cervus elaphus*) [8, 13]. In Sweden and Finland, four and three affected moose were identified, respectively. In Norway, twelve infected moose, 21 reindeer and three red deer were observed. Where cases were detected the prevalence was below 1% [8]. The affected reindeer in Norway were geographically clustered, with 19 cases coming from Nordfjella management zone one. The other two cases were detected in 2020 and 2022 in the Hardangervidda area. In comparison, cases reported in moose and red deer were more geographically scattered [14].

Two main pathological phenotypes can be distinguished in European cervids: the Ly + phenotype characterized by the presence of detectable PrP^Sc^ accumulations in lymphoid tissues, with or without deposits in brain tissue, and the Ly- or sCWD (sporadic CWD) phenotype, with detectable PrP^Sc^ accumulation in the Central Nervous System (CNS), but not in lymphoid tissues [8, 14]. Until now, the Ly + phenotype has been observed in wild reindeer, resembling the patterns described in CWD of North American cervid species. Abundant lymphoreticular involvement reflects the dissemination of prions in the body and natural shedding, which could be associated with a greater probability of natural transmission between live animals [8]. The Ly- or sCWD phenotype has only been observed in old aged moose and red deer. So far, five physio-pathological and biochemical distinct types of cervid TSE, potentially representing different strains, have been identified out of eight European isolates (three reindeer, four moose, one red deer). These putative strains would differ from the North American ones [15–19].

Effective measures to prevent transmission of the disease in cervids are limited. In North America, depopulation of infected farms, double fencing and restrictions on the transport of farmed cervids have not been effective to date [20]. Polymorphisms in the gene encoding the prion protein (*PRNP*) influence susceptibility or resistance to the progression of prion diseases in humans and animals. For example, the selection of *PRNP* alleles associated with resistance to classical scrapie successfully helped controlling TSEs in small ruminant species in several European countries. In North America, the *PRNP* sequence of a dense sampling (i.e. more than 1400 samples) on white-tailed deer showed some protective influence of 95H, 96S, 116G and 226 K [21–23] and 225F for mule deer [24]. For example, in order to reduce the risk of infection, a selective breeding program for farmed white-tailed deer in high-prevalence CWD endemic area, focusing on the elimination of the 96G variant has recently been developed [25]. Increased knowledge of *PRNP* variability in the main European cervid species will make it possible to map frequencies of known *PRNP* alleles and, in the best-case scenario, will allow identification of new alleles that can serve as leverage to combat the spread of CWD. In France, the roe deer (*Capreolus capreolus*) and the red deer are the two most common cervid species. Currently, for these two species, each year the French Biodiversity Agency (OFB) in partnership with the *Fédération Nationale des chasseurs* (national hunters’ federation) and *Fédérations départementales des chasseurs* (departmental hunters’ federations), monitors the hunting tables to estimate the distribution of all wild ungulates present on French territory. These data are based on a system of systematic national surveys centralised and analysed, and serve as a national benchmark. On a national scale, there is no reliable method for correctly estimating the density and/or abundance of free-ranging animals living in the wild in an open area. Depending on the circumstances and the species under consideration, certain methods are preferred to obtain indices and trends on the distribution of animals. However, over long-time scales, knowledge gained from hunting data allows us to assess trends in the abundance and distribution of these animals. For example, in 42 years, hunting quotas have increased by a factor of 8.7 for roe deer and 8.3 for red deer. Roe deer are present in 93 of the 96 departments (all 13 regions) of mainland France, occupying 92% of the territory with one point five to four million individuals. In 2020, red deer were present in 87 departments, occupying an area of 218 177 km^2^, covering almost 40% of the national territory. In 2023, the population was estimated to be in the 150 000 and 400 000 range [26, 27].

The aim of this study was to obtain a reliable picture of the distribution of *PRNP* polymorphisms among red deer and roe deer populations in France. This genetic information will be crucial in estimating the potential susceptibility of these populations to the emergence of CWD.

## Materials and methods

### Animal sampling

For the purpose of this study, a total of 2143 samples belonging to 1134 roe deer and 1009 red deer were collected. This collection of French cervids was obtained thanks to two complementary sampling campaigns referenced as the OFB (French Biodiversity Agency) collection and the INRAE-CEFS (Wildlife Behaviour and Ecology) laboratory collection. In order to obtain a relatively representative sample collection, we tried not to take more than two samples per day from the same hunting area.

### OFB collection

A national sampling of red deer and roe deer populations was carried out during the 2020–2021 and 2021–2022 hunting seasons by the OFB. The sampling plan was forwarded to the departmental hunting federations, which supervised the local hunting societies. In addition, four OFB study areas took part in the sampling. A total of 614 samples of roe deer and 687 samples of red deer were provided. All tissue samples in this collection were sent in a 70% ethanol solution.

### INRAE-CEFS collection

For the regions of France partially or not covered by the OFB programme, additional DNA samples from 520 roe deer and 322 red deer were provided by the INRAE-CEFS laboratory. These were samples collected on live animal as part of a long-term population monitoring programme, or samples collected with the collaboration of national hunting federation and the ELIZ institution (an interdepartmental institution focussed on zoonosis control).

**Figure 1:**
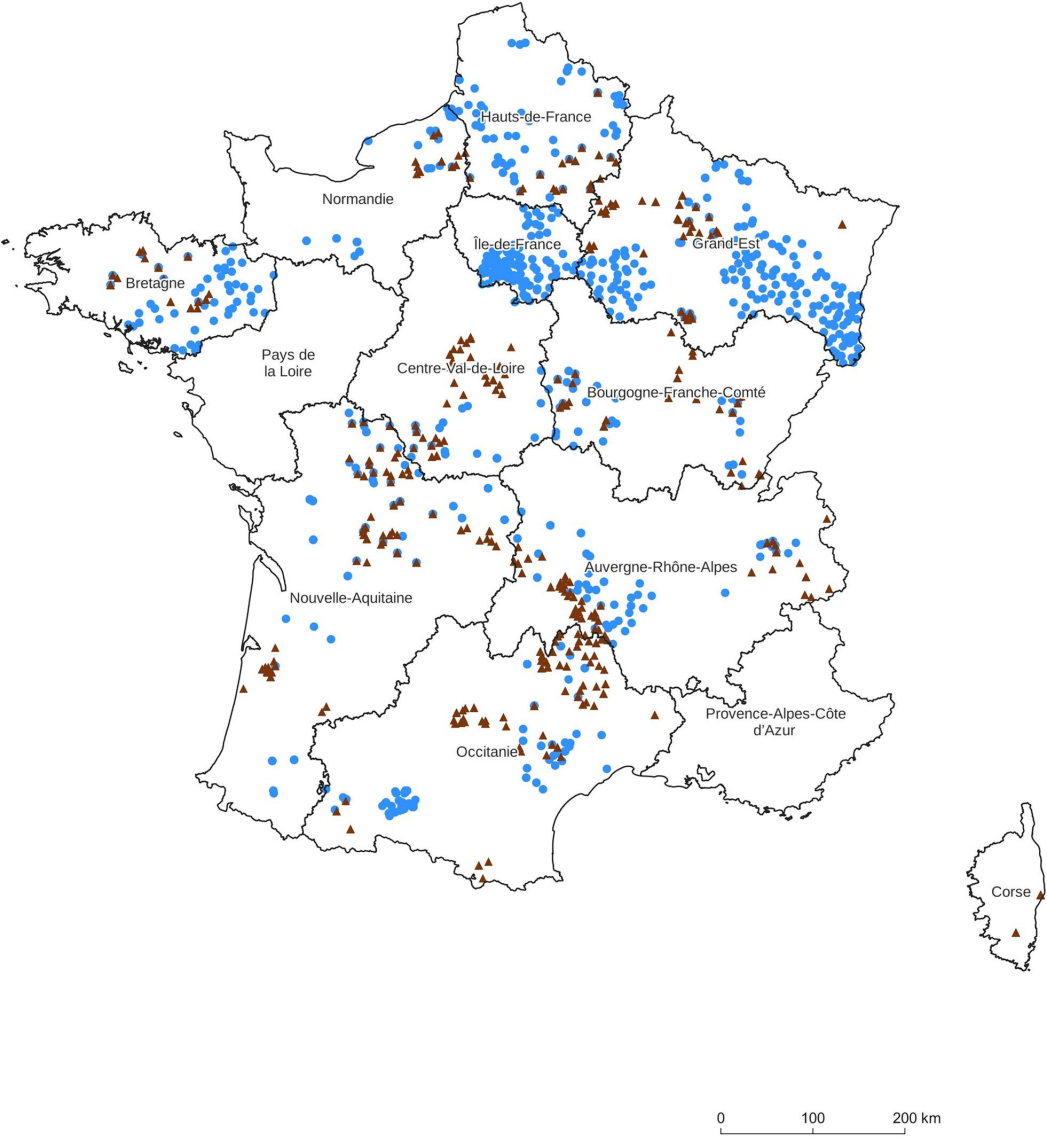
**Geographical distribution of sampling sites in the French regions obtained by the OFB and INRAE-CEFS sampling campaigns**. Each point represents one animal, roe deer (blue circle), red deer (brown triangle).

Overall, samples from the French cervid collection mainly came from hunting (1010 roe deer, 971 red deer). Other samples came from live biopsies (109 roe deer, 38 red deer) or road-kill or slaughtered animals (15 roe deer). Roe deer samples consisted of small sections of muscles (581), spleen (399), skin (118), cartilage (32) and liver (one) and three unspecified. Most were collected between 2020 and 2022 (920) and some (214) between 2004 and 2019. Red deer samples consisted of small sections of muscle (661), skin (229), spleen (93) and cheek (26). Most were collected between 2020 and 2022 (865) and some (144) between 2004 and 2019. For each sample, further information including sex, age class (juvenile, yearling, adult) and coordinates of the municipality of hunting, was collected. The distribution of sampling sites for each species is shown in Figure 1.

To enable a secure sample management and distribution, all the red deer samples (1009) and 75% of the roe deer samples (850) are stored since spring 2024 under the project name CerviFrance at the @BRIDGe Biological Resource Center of the CRB-Anim infrastructure [28].

### Extraction and purification of genomic DNA

A small section of tissue (< 10 mg) was added to 1 mL of lysis buffer (Tris 100 mM pH 7.4, EDTA 5 mM, SDS 0.2%, NaCl 0.2 M) and digested with 12 µL of Proteinase K (20 mg/mL) by incubation for 3 h at 55 °C. After centrifugation for 10 min at 15 000 g, the supernatant was transferred to a new tube and precipitated with 1 mL of isopropanol following a standard precipitation protocol [29]. The DNA was dissolved in 50 or 100 µL of Tris 10 mM pH 7.4 EDTA 0.1 mM buffer at 65 °C during 45 min and stored at −20 °C until use.

For samples (64) not yielding sufficient DNA quality using the above method, an alternative DNA extraction method using Puregene core kit A (158667, Qiagen, Oslo, Norway) was used according to manufacturer’s instructions. The 842 samples from the INRAE-CEFS collection were extracted using the Macherey–Nagel Nucleospin Tissue kit (Cat. # 740952.250) as per manufacturer’s instructions.

### *PRNP* gene amplification and sequencing

The open reading frame (ORF) of cervid *PRNP* (771 bp) was amplified using Go Taq G2 flexi-PROMEGA polymerase and buffers, in a 50 µL of reaction volume containing 100 µM of each dNTP (Promega), 3 mM of MgCl2, 10 µmol of each oligonucleotide primer (Eurofins Genomics, Ebersberg, Germany), 1.5 U of Taq polymerase and 100 to 150 ng of genomic DNA. The PCR forward primer was ACA CCC TCT TTA TTT TGC AG (Ce-08-F) and the reverse primer was AGA AGA TAA TGA AAA CAG GAA G (Ce-778-R). PCR conditions were at 94 °C for 5 min, followed by 40 cycles of 94 °C for 30 s, 58 °C 30 s and 72 °C for 1 min, and a final extension at 72 °C for 3 min.

PCR products (830 pb) were visualized electrophoretically on a 1.5% agarose gel before being sent for a double strand sequencing with Primers Ce-08-F and Ce-778-R to Eurofins Genomics (Ebersberg, Germany). Chromatographs were checked and analysed using CodonCode Aligner [30].

### Cloning

*PRNP* coding sequences were amplified as described in the *PRNP* gene amplification and sequencing section, but with a final extension at 72 °C for 7 min. The amplicons were purified using PURELINK Quick PCR purification Kit (K31001, Invitrogen, Vilnius, Lithuania) according to manufacturer’s instructions. Purified products were then cloned using TOPO TA cloning Kit (K450040, Invitrogen, Vilnius, Lithuania) following the procedure provided by the manufacturer. For each cloned sample, two to four clones were selected and sent to Eurofins Genomics (Ebersberg, Germany) for both strand sequencing with primers Ce-08-F and Ce-778-R. Chromatographs were checked and analysed using CodonCode Aligner [30].

### Species verification

To control the correct assignment of the species indicated in our database with each DNA sequenced**,** we checked all the positions which were different between roe deer and red deer sequences, namely positions 63 (codon 21 g\c), 221 (codon 74 a\t), 384 (codon 128, t\c), 408 (codon 136, c\t), 438 (codon 146, c\t), 618 (codon 206, t\c) and 741 (codon 247, a\c).

### Maps and statistical analysis

The map with the geographical distribution of roe deer and red deer genotyped samples (Figure 1) was generated using QGIS [31].

The linkage disequilibrium (LD) between pairs of positions was measured by the so-called r2 coefficient [32]. This measure is simply the square of the conventional correlation of gene frequencies in the sample (Table 5).

Independence between pairs of positions was tested with the Fisher’s exact test (Table 5) [33].

Residual-based shading plots, or mosaic plots were used to visualize contingency tables [34]. In these plots, the area of the cell represents the counts, the cell’s width represents the marginal probability within the row, while the cell’s height represents the marginal probability within the column. Under the null hypothesis of independence, all the cells are grey. The colour code in the legend represents the standardized Pearson’s residuals from independence and shows whether the number of observed individuals is greater (blue) or smaller (red) than that theoretically expected.

**Figure 2:**
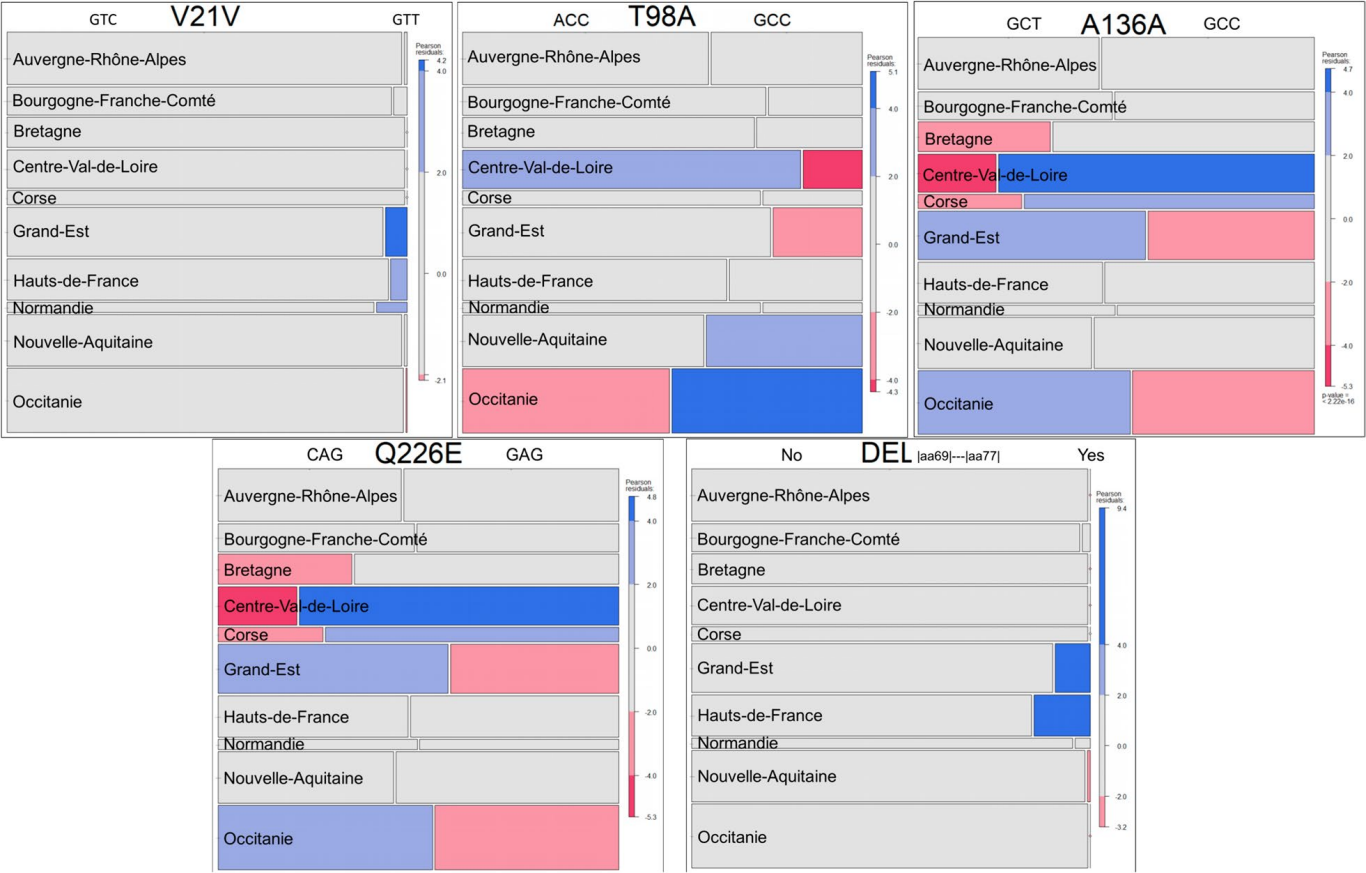
**Mosaic diagram showing the contingency tables for each SNP-region combination.** The area of the cell (rectangle) represents the quantities (numbers), the width of the cell represents the marginal probability in the row (region), the height of the cell represents the marginal probability in the column (number of animals per SNP within a region) and the colour of the cell represents the Pearson standardised residual with respect to independence (blue numbers higher than expected and red numbers lower than expected). The graphs were coloured on the basis of the Pearson residuals.

**Figure 3:**
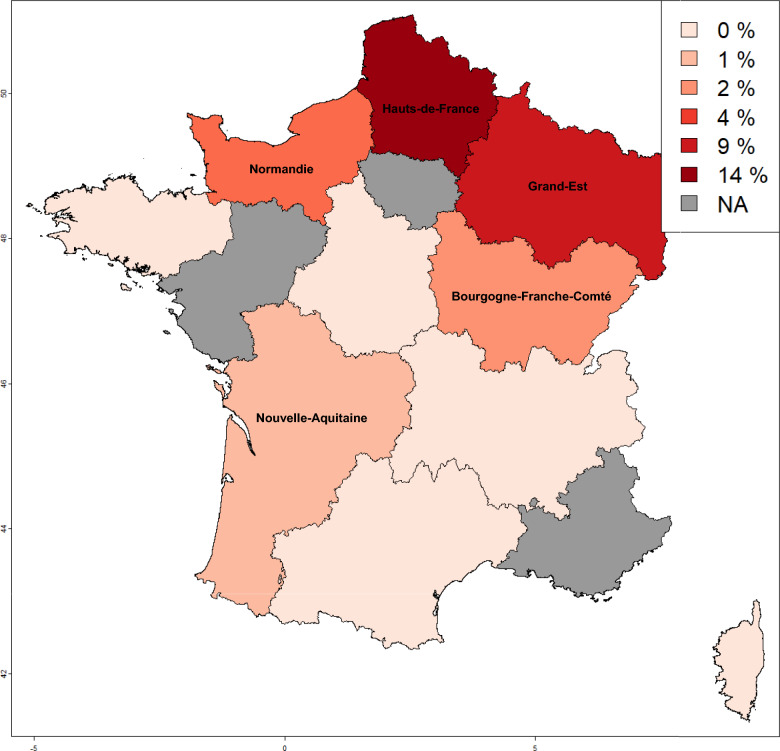
**Geographical localisation ∆69-77 allelic frequencies obtained in red deer in France.** The map has been coloured according to the frequencies. NA: Not Applicable.

**Figure 4:**
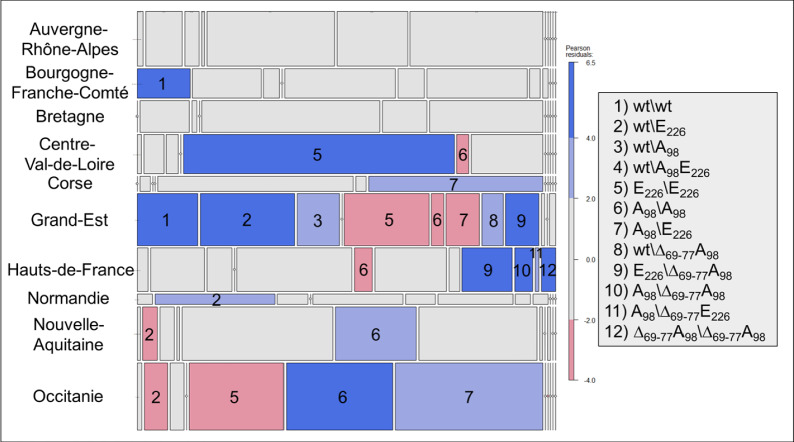
**Mosaic diagram showing the contingency tables for each Genotype-region combination.** The area of the cell (rectangle) represents the quantities (numbers), the width of the cell represents the marginal probability in the row (region), the height of the cell represents the marginal probability in the column (number of genotypes in a region) and the colour of the cell represents the Pearson standardised residual with respect to independence (blue numbers higher than expected and red numbers lower than expected). The graphs were coloured on the basis of the Pearson residuals.

Statistical analyses (Tables 2, 3, 4) and plots were realized with the R package (R Core Team, 2003). Mosaic plots were realized with the vcd package (Figures 2 and 4) [35]. The Map corresponding to Δ_69-77_ allelic frequencies was done with the geodata [36] and terra [37] packages (Figure 3).

### Accession numbers

Seven sequences were deposited in GenBank with the following accession numbers: PP512526 (red deer with A_98_ haplotype), PP512527 (red deer with A_98_E_226_ haplotype), PP512529 (red deer with Δ_69-77_A_98_ haplotype), PP512532 (red deer with E_226_ haplotype and synonymous mutation at codon 136), PP512533 (red deer with haplotype E_226_ haplotype and synonymous mutations at codons 21 and 136), PP512535 (wildtype red deer) and PP512539 (roe deer A_37_ haplotype).

## Results

### Sampling

After extraction of genomic DNA, amplification, sequencing and analysis of the cervid *PRNP* ORF, we obtained sufficient readable sequences for 1116 roe deer out of the 1134 animals collected and 998 red deer out of the 1009 animals collected (i.e. an efficiency of 98%). The geographical location of these animals is shown in Figure 1. The number of samples per species and per region is shown in Table 1. 53% and 45% of the samples were females for red deer and roe deer, respectively. This collection met with a certain number of criteria such as historical origin, spatial distribution, sex ratio and geographical distance between animals.

**Table 1:** **Distribution by region and sex of roe deer and red deer sequenced for** ***PRNP* in the present study**

	Roe deer				Red deer			
	Number of individuals	Male	Female	Not specified	Number of individuals	Male	Female	Not specified
Auvergne-Rhône-Alpes	83	42	39	2	138	69	69	
Bourgogne-Franche-Comté	65	35	30	0	74	42	32	
Bretagne	84	46	38		79	39	40	
Centre-Val-de-Loire	40	23	17		101	42	54	5
Corse	/	/	/		38	20	18	
Grand-Est	283	129	99	55	129	54	74	1
Hauts-de-France	118	61	57		109	40	69	
Ile-de-France	100	56	44		/	/	/	
Normandie	51	24	27		26	16	10	
Nouvelle-Aquitaine	173	95	74	4	135	65	68	
Occitanie	119	61	53	5	169	79	90	2
Total	1116	572	478	66	998	466	524	8

Identified non-synonymous polymorphisms were reported as variations from a consensus or wild type sequence (wt, roe deer and red deer T_98_P_168_Q_226_). We followed the EFSA (2023) suggestions on naming alleles by codon position and a one letter amino acid abbreviation for all positions that deviate from wt.

### Roe deer

All analysed French roe deer (*n* = 1116) were homozygous wt at the *PRNP* locus, except one heterozygous animal with a non-synonymous mutation at codon 37 (g/c position 110) resulting in a glycine to alanine amino acid change (G37A). The presence of this substitution was confirmed by three independent PCR amplifications of the ORF and subsequent sequencing in both strands. The amino acid sequence of all other animals showed 100% identity with the cervid consensus sequence. Except for one animal, this gene appears to be fixed.

### Red deer

In red deer samples (*n* = 998), we detected polymorphisms at five nucleotide positions (SNPs) and one novel deletion of 24 bp. Three synonymous substitutions were identified at codon 21 (c/t, nucleotide position 63), 78 (g/a nucleotide position 234) and 136 (t/c nucleotide position 408). The substitution at codon 78 was only present in three individuals among the 998 studied. The substitutions at codon 21 and 136 were present at frequencies of 2% and 45%, respectively (Table 2). Two non-synonymous substitutions were observed at codon 98 (a/g nucleotide position 292) and 226 (c/g, nucleotide position 676), giving rise to threonine to alanine (T98A) and glutamine to glutamic acid (Q226E) amino acid changes, respectively. At codon 98, the wt allele was the most common with a frequency of 68%, whereas at codon 226, the two alleles had more balanced frequencies (45% for Q, 55% for E). A new 24 bp deletion was identified within the octa-peptide repeat region with a frequency of 3%. This ccaacctcatggaggtggctgggg deletion corresponds to the deletion of codons 69 to 77, QPHGGGWG (Δ_69-77_, position 207 to 230). Except for the deletion Δ_69-77_, observed polymorphisms have been previously documented in European red deer.

**Table 2:** **Allele frequencies of the * PRNP* polymorphisms in red deer from France (*n*** **= 998)**

Codon	Amino acid variation	Nucleotide sequence	Frequency (%)
21	V	GTC	98
	V	GTT	2
98	T	ACC	68
	A	GCC	32
136	A	GCT	45
	A	GCC	55
226	Q	CAG	45
	E	GAG	55
Δ_69-77_		Not present	97
Δ_69-77_		Present	3

For each codon, the wildtype sequence is shown at the top and the mutated sequence at the bottom.

We found significative variations between French regions in the frequency of the substitutions identified (Figure 2, Table 3). The frequency of the A_98_ allele was significantly lower in the Centre Val-de-Loire region (15%) and in the Grand-Est (22%), whereas it was higher than expected in Occitanie (48%). Furthermore, five regions-Bretagne, Centre Val-de-Loire, Corse, Grand-Est and Occitanie-differed significantly in terms of frequencies of alleles at positions 136 and 226. The E_226_ allele was significantly more frequent in the Centre Val-de-Loire and Corse regions, with frequencies of 80% and 74%, respectively, whereas it was below the expected value in the Grand-Est and Occitanie regions (42% and 46%, respectively). The Δ_69-77_ deletion was detected in the regions of Normandie, Hauts-de-France, Grand-Est, Bourgogne-France-Comté and Nouvelle Aquitaine (Figure 3). This deletion was mainly found in regions bordering Germany, Belgium and Luxembourg, with frequencies of 9% and 14% for the Grand-Est and Hauts-de-France regions, respectively.

**Table 3:** **Percentage of allele frequencies of * PRNP* polymorphisms in French red deer in 10 regions of France (** ***n*** **= 998)**

Codon	21		98		136		226		Δ_69-77_	
Amino acid variation	V	V	T	A	A	A	Q	E		
Nucleotide sequence	GTC	GTT	ACC	GCC	GCT	GCC	CAG	GAG	Not present	present
Auvergne-Rhône-Alpes	99	1	62	38	46	54	46	54	100	0
Bourgogne-Franche-Comté	97	3	76	24	49	51	49	51	98	2
Bretagne	100	0	73	27	34	66	34	66	100	0
Centre-Val-de-Loire	100	0	85	15	20	80	20	80	100	0
Corse	100	0	75	25	26	74	26	74	100	0
Grand-Est	95	5	78	22	58	42	58	42	91	9
Hauts-de-France	96	4	67	33	47	53	48	52	86	14
Normandie	92	8	75	25	50	50	50	50	96	4
Nouvelle-Aquitaine	99	1	61	39	44	56	44	56	99	1
Occitanie	100	0	52	48	54	46	54	46	100	0
*p*-value	0.0001		0.0001		0.0001		0.0001		0.0001	

The *p* values are based on the Fisher’s exact tests performed on the corresponding contingency tables.

### Haplotype and genotype frequencies in red deer

Of the 998 red deer sequenced, 315 had multiple polymorphisms at non-synonymous substitutions (Δ_69-77_, codons 98 or 226). To determine the haplotype of these animals, their amplified ORFs were cloned and sequenced and three of them were excluded due to inconclusive sequence results. Haplotype frequencies were therefore calculated on a total of 995 animals (Table 4). Among the eight possible haplotypes we have detected six haplotypes namely wt (T_98_Q_226_), E_226_, A_98_, A_98_E_226,_ Δ_69-77_A_98_ and Δ_69-77_E_226_. The deletion carried by 55 animals appeared, with the exception of one animal, associated with A_98_.

**Table 4:** **Haplotype frequencies of* PRNP* non-synonymous polymorphisms and Δ**_**69-77**_** deletion in French red deer populations (*n* = 995)**

Haplotype	Frequency (%)
wt	12.75
E_226_	55.2
A_98_	28.9
A_98_E_226_	0.1
Δ_69-77_-A_98_	3
Δ_69-77_-E_226_	0.05

Haplotype frequencies revealed that the most prevalent haplotype was E_226_ with a frequency of 55.2% while the wt haplotype was present at a frequency of 12.75% (Table 4). The two newly identified novel haplotypes carrying the deletion were relatively rare (3% for Δ_69-77_ A_98_ and 0.05% for Δ_69-77_ E_226_).

Pairwise analysis of linkage disequilibrium (LD) between positions 63, 292, 408, 676 and Δ_69-77_ (codons 21, 98, 136, 226 and Δ_69-77_) indicated that these positions were almost all genetically linked (Table 5). However, at positions 63 (codon 21) no genetic association was found with the position 292 (codon 98) neither with the deletion (r2 = 0 and *P*-value = 0.025 and 0.61 respectively, Table 5). These analyses involved only 889 samples as 106 animals with multiple polymorphisms at synonymous substitutions were not cloned.

**Table 5:**
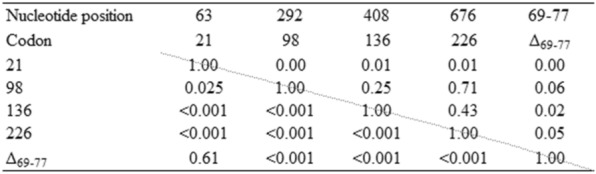
**Linkage disequilibrium (LD) between position pairs of** ***PRNP* variant and dependence between** ***PRNP* position pairs**

The r2 Coefficients appear above the diagonal and the *p*-values based on Fisher’s exact tests are below the diagonal.

We obtained a total of twelve genotypes. Four were rare, having a frequency of less than 1%. The most frequent genotypes were E_226_\E_226_ and A_98_\E_226_ respectively (Table 6). Overall, similar frequencies were observed between heterozygous (48%) and homozygous (52%) animals in the French population, although the deletion was more frequent at heterozygous state (89%).

**Table 6:** **Genotype frequencies of * PRNP* polymorphisms in French red deer populations (*n*** **= 995)**

Genotype	Frequency (%)
wt\wt	4
wt\E_226_	11.2
wt\A_98_	4.6
wt\A_98_E_226_	0.2
E_226_\E_226_	35
A_98_\A_98_	12.9
A_98_\E_226_	26.6
wt\Δ_69-77_-A_98_	1.4
E_226_\Δ_69-77_-A_98_	2.7
A_98_\Δ_69-77_-A_98_	0.7
A_98_\Δ_69-77_-E_226_	0.1
Δ_69-77_-A_98_\Δ_69-77-_A_98_	0.6

We performed a mosaic plot based on genotypes obtained from 995 animals to provide a regional scale characterization of their frequencies (Figure 4). The number of genotypes observed per region varied from four in the Corse region to eleven in the Hauts-de-France region. Although each region had its own particularities, it can be seen that the Grand-Est region differed the most, with eight genotypes having a frequency significantly different from those observed on average in France. In this region, genotypes wt\wt, wt\E_226_, wt\A_98_, wt\Δ_69-77_-A_98_ and E_226_\Δ_69-77_-A_98_ were significantly more frequent than expected while E_226_\ E_226_, A_98_\A_98_ and A_98_\E_226_ were significantly less. The difference observed in the Hauts-de-France region was mainly due to the genotype associated with the deletion.

## Discussion

This study is the first investigating the diversity of the *PRNP* gene ORF in the two most common cervid species in France. A total of 2114 animals were studied, 1116 roe deer and 998 red deer. The nature of this sampling makes it as representative as possible of these two species in France. As such, this collection will be preserved for future genetic studies by the @BRIDGe Biological Resource Center of the CRB-Anim infrastructure. This study represents the largest survey of *PRNP* genetic variation in roe deer and red deer population of any European country to date.

Except for one animal, all the 1116 French roe deer were monomorphic in *PRNP* sequence, with 100% amino acid sequence identity with the cervid wt PrP. Similar observations were reported in roe deer from Great Britain (*N* = 297), Alpine arc of Italy (*N* = 189), Northeast of Spain (*N* = 44), Sweden (*N* = 11) or Norway (*N* = 46) [38–41]. These results are consistent with the history of colonization of Europe by roe deer. The European roe deer is one of the most common ungulates in Europe. It is distributed from the Mediterranean zone to Scandinavia and the eastern border of its range reaches western Russia [42, 43]. This species experienced dramatic fluctuations due to climatic factors and anthropogenic influences. It was forced into refugia to the Mediterranean peninsulas with the exception of south-western France and the surroundings of the Carpathian during the Last Glacial Maximum (LGM, 21.0–14) [44]. During postglacial periods the species distribution extended further north and on western, central and northern Europe around 9600 years ago. More recently between the 17th and early twentieth century, extensive deforestation, poaching and excessive hunting caused a dramatic decline of the species throughout Europe with even local eradication in central and south Iberia, in western Italian alps and Apennines and in Greece [45]. After the Second World War, for example, concerted management structures or conservative harvesting rules were set up at national levels and helped to increase the number of individuals of this species.

Recently a phylogeographic study based on the analysis of 3010 control mitochondrial DNA sequences from European roe deer have showed a strong geographical pattern with a clear division into three major clades: Eastern, Western and Central. This latest clade covered large parts of the continent. In addition, these data suggested that the refugial population of southern France might have spread to cover the whole western and north-western Europe, possibly reaching the central and eastern parts of the continent [43].

In our study, red deer showed greater *PRNP* sequence variation with two non-synonymous substitutions (T98A; Q226E), three synonymous substitutions (codons 21, 78 and 136) and one novel deletion of 24pb (Δ_69-77_). These substitutions have been previously documented in European red deer and the deletion Δ_69-77_ has also been recently detected in Germany [46]. After cloning *PRNP* sequences from animals presenting multiple non-synonymous polymorphisms, we found a significant linkage between SNPs resulting in substitutions at positions 98, 136, 226 and the deletion. We identified six haplotypes, three of which are more frequent and correspond to those found mainly in Europe. However, it should be noted that the number of European red deer studied in different countries at the level of *PRNP* is uneven and represents a different power of resolution. Overall, less than 1200 animals have been previously surveyed: 627 in Great Britain, 209 in north-eastern Spain, 191 in Italy, 106 in 40 Norwegian municipalities and 55 in central-eastern Portugal [38, 40, 41, 47–49]. In the present study, the wt haplotype was relatively rare as reported in Norway (11%), whereas it was observed across Europe at frequencies ranging from almost 30% (England, Scotland) to 71% (Portugal). Haplotype E_226_ had a frequency similar to that observed in Scotland (50%), whereas it was found to be very common in England and Norway and only moderately in the other European countries analysed. Haplotype A_98_ (28.9%) was present at similar frequencies to those of Spain and Czechoslovakia, whereas it was not detected in England.

Although based only on a single gene, these results were consistent with broad European red deer phylogeographic studies which classified extant European red deer into five mitochondrial lineages. Among these lineages, the western haplogroup (designated A) was distributed along a south-north axis from Iberia through France and the British Isles, to Scandinavia and Central Europe [50–53]. An eastern haplogroup (designated C) was found in the Balkans and parts of Eastern and Central Europe. These are the two major lineages. They co-occur in, for example, Czechoslovakia, Austria and Poland [53, 54].

In France, red deer was historically abundant until the early 18th century, then declined sharply with the democratisation of hunting rights during the French Revolution and poaching, which was accelerated by the proliferation of weapons during and after the wars of 1914–1918 and 1939–1945, and the need of food during the wars. After this period, the red deer slowly recovered, thanks to protection laws, hunting reserves and restocking. It has been estimated that around a third of all populations were artificially established between the 1950s and 1970s with the release of red deer mainly from the Domaine National de Chambord enclosure and with animals from the Petite-Pierre National hunting and wildlife Reserve. In 2000, it was estimated that the proportion of red deer resulting from these artificial reintroductions reached 50% of the national population [55]. This repopulation was also accompanied by concerted management structures, conservative harvesting rules (generalisation of hunting plans in 1979), the abandonment of agricultural land to fallow but also the increase of forest areas and the extinction of natural predators. However, this expansion has been highly variable, fluctuating in response to the local hunting laws or forestry demands [55–57]. A parallel could be drawn between the origin of the deer populations present in France and the distribution pattern of *PRNP* haplotypes. For example, the most frequent haplotype in France, E_226_, corresponded to the genotype in excess in the Centre-Val-de-Loire region, of which the Domaine of Chambord is a part. In addition, Corse red deer, which are currently protected, showed a frequency of the A_98_\E_226_ genotype that was significantly different from that expected in France, and this region and Bretagne both showed least *PRNP* diversity. These results were consistent with previous studies based on microsatellite data, where a reduced level of genetic variability had been observed in red deer from four forests in Bretagne [57]. It is plausible to explain this low genetic variability by a combination of geographic isolation and a small population size in the recent past. In fact, the original Corse red deer population disappeared at the end of the 1960s due to a major opening up of its habitat, uncontrolled hunting and intensive poaching. This population was then re-established through releases of 300 Sardinian animals considered to be of the same origin, but the effective population size in Sardinia populations has been estimated at 8, which is particularly low [26, 50, 58].

Finally, the region with the greatest number of different *PRNP* genotypes was the region of Grand-Est which includes the Petite-Pierre reserve. Additionally, a recent study based on mitochondrial DNA highlighted that in north-eastern France red deer populations were built from a few hundred individuals that have subsisted in remote valleys of the Vosges mountains [56]. This particular history might explain the detection of the rare Δ_69-77_ allele that is also found in Germany [46].

Our study has provided an analysis of sequence variation in the *PRNP* ORF in the two most common cervid species in France, the roe deer and the red deer. As in other parts of Europe, we found genetic homogeneity in the French roe deer and greater diversity with regional differences in the red deer. To date, a total of 35 polymorphic codons have been reported in the *PRNP* ORF in cervid species, with the greatest number of polymorphisms observed in white-tailed deer, sika deer (*Cervus nippon*) and reindeer. For example, in a recent study of 221 wild Norwegian reindeer, the 225Y allele was associated with reduced susceptibility to CWD compared with the wt and del_84-91_ alleles, with wt/wt animals showing the highest susceptibility [38]. Thus, overall, the protective influence of the different alleles has been highlighted, but so far it appears that all *PRNP* genotypes reported in cervids are affected by CWD [59, 60]. One of the original findings of our study is the identification of a new allele presenting the deletion of an octa-peptide repeat region (OR) in red deer. The physiological and pathological roles of this OR region on PrP^c^ are still understudied. The prion gene family derived from a subset of the ZIP family of metal ion transporters [61]. PrP^c^ is involved in different functions, which include among others critical roles in the maintenance of metal (copper, zinc, iron, manganese) homeostasis. Metals are essential for normal brain functions; their concentrations and chemical forms are strictly regulated and their dysregulation is linked to several neurodegenerative diseases [62]. Metals are known to crosslink proteins by binding to several amino acids such as histidine, arginine and phosphorylated amino acids. PrP^c^ is more selective for Cu^2+^ compared to other metals due to the metal chelating effects of histidine [63]. PrP^c^ possesses six histidine residues, including one in each of its OR. This OR region could act as a conformational switch and be involved in prion infection [64]. Disruption of the integrity of this region by the insertion or deletion of the OR sequence have been shown to affect the characteristic of resulting oligomers and fibrils as well as disease phenotypes, in different clinical, biophysical, in vitro or transgenic mouse studies [65]. For example, it has been shown that the number of ORs can be inversely associated with incubation times after BSE prion inoculation into transgenic mice expressing a bovine PrP [66–68]. Furthermore, using OR deficient PrP^c^ mice, it has been recently suggested that the OR region might be involved in prion pathogenesis in a strain dependent manner [69, 70]. Thus, investigations of the properties of the newly identified PrP_Δ69-77_ using different panel of CWD isolates from Scandinavia and North America are worth undertaking and are indeed currently being initiated.

It remains that the first cases of cervids affected by CWD discovered in Scandinavia are a warning to other European countries which, on the basis of the genotypes observed to date, would appear to be rather susceptible to CWD. The appearance of TSE cases, particularly in geographical areas not currently sampled or tested, cannot be ruled out in European cervids and measures must be taken as soon as possible to avoid the spread of this disease in target populations, as it has unfortunately been observed in North America. There are many complex rules and procedures governing this wildlife disease, so future monitoring at a European-wide level will require multidisciplinary approaches.

## Data Availability

The @BRIDGe Biological Resource Center of the CRB-Anim infrastructure will manage and distribute the CerviFrance collection [28]. The datasets used and/ or analyzed during the current study are available from the corresponding author on reasonable request.

## Abbreviations

CWD: chronic wasting disease
PRNP: prion protein gene
TSE: transmissible spongiform encephalopathy
PrP^C^: protease-sensitive cellular prion protein
PrP^Sc^: abnormal protease-resistant isoform of the prion protein
US: United States
Ly: lymphoid
sCWD: sporadic chronic wasting disease
CNS: central nervous system
TSEs: transmissible spongiform encephalopathies
i.e.: *id est* (That is)
OFB: French Biodiversity Agency
CEFS: Wildlife Behaviour and Ecology
INRAE: National research institute for agriculture, food and the environment
ORF: open reading frame
LD: linkage disequilibrium
wt: wild type
SNPs: single nucleotides polymorphisms
PrP: normal cellular prion protein
LGM: last glacial maximum
OR: octa-peptide repeat region
ZIP: zinc-regulated transporters, iron-regulated transporter-like protein
